# Completion of Proteomic Data Sets by K_d_ Measurement Using Cell-Free Synthesis of Site-Specifically Labeled Proteins

**DOI:** 10.1371/journal.pone.0082352

**Published:** 2013-12-10

**Authors:** Paul Majkut, Iris Claußnitzer, Helmut Merk, Christian Freund, Christian P. R. Hackenberger, Michael Gerrits

**Affiliations:** 1 Department Chemical Biology II, Leibniz-Institut für Molekulare Pharmakologie (FMP), Berlin, Germany; 2 RiNA GmbH, Berlin, Germany; 3 Institut für Chemie und Biochemie, Freie Universität Berlin, Berlin, Germany; 4 Institut für Chemie, Humboldt-Universität zu Berlin, Berlin, Germany; Hungarian Academy of Sciences, Hungary

## Abstract

The characterization of phosphotyrosine mediated protein-protein interactions is vital for the interpretation of downstream pathways of transmembrane signaling processes. Currently however, there is a gap between the initial identification and characterization of cellular binding events by proteomic methods and the in vitro generation of quantitative binding information in the form of equilibrium rate constants (K_d_ values). In this work we present a systematic, accelerated and simplified approach to fill this gap: using cell-free protein synthesis with site-specific labeling for pull-down and microscale thermophoresis (MST) we were able to validate interactions and to establish a binding hierarchy based on K_d_ values as a completion of existing proteomic data sets. As a model system we analyzed SH2-mediated interactions of the human T-cell phosphoprotein ADAP. Putative SH2 domain-containing binding partners were synthesized from a cDNA library using Expression-PCR with site-specific biotinylation in order to analyze their interaction with fluorescently labeled and *in vitro* phosphorylated ADAP by pull-down. On the basis of the pull-down results, selected SH2’s were subjected to MST to determine K_d_ values. In particular, we could identify an unexpectedly strong binding of ADAP to the previously found binding partner Rasa1 of about 100 nM, while no evidence of interaction was found for the also predicted SH2D1A. Moreover, K_d_ values between ADAP and its known binding partners SLP-76 and Fyn were determined. Next to expanding data on ADAP suggesting promising candidates for further analysis *in vivo*, this work marks the first K_d_ values for phosphotyrosine/SH2 interactions on a phosphoprotein level.

## Introduction

The identification and characterization of protein-protein interactions constitutes a corner stone for the understanding of signaling pathways and other cellular processes. Signaling pathways comprise a combination of directed series of protein-protein interactions that participate in larger protein interaction networks [1,2]. Typically, such protein interactions are mediated by modular binding domains of limited size that recognize specific sequence motifs on partner proteins. This can be seen for example if protein interactions are triggered by posttranslational modifications (PTMs), in which case the modified sequence motifs are recognized by conserved binding entities, such as 14-3-3, MBT, PTB or SH2 domains, that are able to participate in a largely diverse set of possible interactions [3–5].

While the assembly of protein networks depends on many factors such as expression level, modification state and subcellular localization of the proteins, the basic unit is the distinct interaction between two proteins. The strength of such an interaction is described by the equilibrium rate constant (K_d_). Knowing the K_d_ of a protein-protein interaction is important in order to establish binding hierarchies within the framework of pathways and to feed predictive models, especially when regarding the wide-ranging specificity of modular binding domains such as SH2 [4]. Hence, the full characterization of protein-protein interactions requires both, a qualitative analysis of individual binding events in a physiological context and a quantitative determination of K_d_ values.

Nowadays, particularly MS-based techniques allow the identification and characterization of a huge number of interacting proteins even in complex biological samples [6,7]. On the other hand, equilibrium rate constants are commonly determined using widespread techniques such as surface plasmon resonance (SPR) [8], isothermal titration calorimetry (ITC) [9,10] and microscale thermophoresis (MST) [11,12]. 

However, the expansion of proteomic interaction data into quantitative binding information on a biophysical level remains still an obstacle in particular since it requires the availability of protein interaction partners in sufficient quality and quantity. The synthesis of the required proteins can be tedious and limiting downstream analysis, especially when addressing multiple possible interaction partners at the same time. In addition, modified interaction partners are often hard to obtain at a comparable modification degree and sufficient quantity. A good example for that is the group of tyrosine phosphorylated proteins. These limitations strengthen the need to precisely co-ordinate protein synthesis, validation of interacting proteins and method of K_d_ measurement. In this regard the number of steps between template generation, protein synthesis and K_d_ determination should be minimized as well as the consumption of protein. In addition, it is an advantage to filter eligible direct interactions prior to K_d_ measurement in order to focus on relevant interactions especially when regarding large proteomic data sets.

Quantitative microarrays represent an alternative to probe direct interactions by type of a cross control of proteomic approaches. They have been used to identify biochemical interactions *de novo* and to generate K_d_ values of the interactions between isolated SH2 domains and short phosphopeptides derived from distinct phosphorylation sites of the target protein [4]. However, the *de novo* generation of microarrays is a battle of material. They are also oversized when only a limited set of pre-selected interactions is to be further analyzed. Furthermore, drying of microarrays can affect the folding state of the equipped proteins influencing their ability to interact.

Bead-based binding assays, available for example in the form of magnetic nanoparticle pull-down, are a cost-effective and flexible alternative to microarrays [13,14]. They get by on low protein amounts like in microarrays. In addition, they provide an inherent spatial separation of proteins and are generated with freshly synthesized proteins using liquid handling. Thus the number of samples can easily be adapted to the preliminary results of proteomic studies. Recently, even a method for K_d_ estimation based on pull-down was described [15]. Therefore, pull-down should be well suited to confine the number of contemplable interactions and to perform a ranking of interactions based on relative affinities prior to their proper K_d_ determination.

In this study, we present a straightforward approach to expand proteomic data sets by K_d_ values in order to predict binding hierarchies. Hereby, we aimed to simplify the whole process from template generation to the validation of biochemical interactions up to the read-out of K_d_ data.

Our strategy combines the cell-free synthesis of a limited set of interaction partners (10 to 20) together with the target protein and the analysis of their interaction by pull-down [15,16] and MST [11,12] into a co-ordinated and new approach. Pull-down is thereby used to select binding partners based on their relative affinities before the K_d_ measurement itself is performed using MST. Here it was important for us that both methods pull-down and MST rest upon sensitive protein detection by fluorescence. This allows to get on with small protein amounts minimizing protein consumption and to use the same fluorescent protein for all analysis steps. We chose the SNAP-tag [17,18] as a tool for fluorescence labeling as it enables the site-directed labeling of larger proteins as well as small peptides in the form of fusion proteins in cell-free protein synthesis reactions. Combining Expression PCR starting from a cDNA vector collection [19] with site-specific biotinylation [20] offered us a fast access to multiple putative binding partners for high-affinity immobilization on magnetic beads. This permits us to shorten the way to pull-down by omitting cloning steps prior to cell-free protein synthesis and purification steps prior to immobilization allowing an accelerated and more flexible response to the results of preceding proteomic studies.

We focused on interactions of the human T-cell adhesion and degranulation promoting adapter protein (ADAP) with SH2 domain containing proteins. ADAP is known to be multiply phosphorylated on tyrosine residues by Fyn kinase, particularly on Tyr 595, Tyr 625 and Tyr 651 [21,22], which lead ADAP to interact with SLP-76 [23], NCK1/2 [24] and Fyn [25,26] via their SH2 domains. This renders ADAP to act as a hub in T-cell mediated signaling cascades where it is thought to couple TCR stimulation with integrin activation by mediating increased integrin avidity [27–29]. Previously, a proteomic approach revealed twelve further possible SH2-domain containing ADAP interactors using SILAC combined with pull-down on solid-phase derived phosphopeptides in Jurkat T-cell lysates [30]. Among them were SH2 proteins that are normally attributed to other pathways, for example PIK3R1, which regulates cell growth and cytoskeletal rearrangement [31] and Rasa1, which feeds into the MAPK cascade and controls proliferation and motility [32]. So far no connection of ADAP signaling to these pathways within a larger protein interaction network has been reported.

SH2 domains are referred to recognize rather small motifs (residues -2 to +5 relative to pTyr) [33–36]. Although an individual SH2 shows preference for certain amino acid residues, the same peptide sequence can bind to many SH2 domains with reasonably significant affinity [4,37]. So far, viable K_d_ values of SH2 domain-mediated interactions are usually measured based on pTyr-containing synthetic peptides covering discrete phosphorylation sites of the target proteins [37,38]. K_d_ values are not at all available for ADAP. Our goal in this study was to distinguish putative binding partners of ADAP on a protein level. In particular, we asked how much the above-mentioned ADAP interactions differ in their K_d_ values in order to predict possible binding occupancies for ADAP in the context of a more complex protein interaction network. This question is particularly interesting when keeping in mind that more than 10,000 pTyr sites [39] of the human proteome compete for 121 different SH2 domains [40]. 

## Methods

### Synthesis of SH2 proteins

Linear DNA templates were obtained via a two step PCR starting from a cDNA library. In the first PCR step, gene specific primers (Table S3) were used to amplify the open reading frame (ORF) of the respective gene. Hereby a constant sequence containing an *amber* stop codon at position 2 was added to the orf. Regulatory elements necessary for efficient translation (T7 promoter, ribosome binding sites etc.) were added during the second PCR step using a primer mixture provided with the Linear Template Generation Kit ssp Label (RiNA GmbH, Berlin, Germany). SH2 protein synthesis with site-directed incorporation of biotinylated lysine was performed using an *E. coli*-derived RF1-depleted cell-free system [20] supplemented with 12 µM BioLys-tRNA_CUA_, i.e. amber suppressor tRNA charged with biotinylated lysine. A corresponding system is available as Site-Specific Biotin Kit at RiNA. Reactions were incubated for 45 min at 30°C on an Eppendorf Thermomixer at 500 rpm. Protein yields were quantified via TCA-precipitation and scintillation counting based on the incorporation of ^14^C-Leu (Perkin-Elmer) added to the cell-free reactions as a tracer. Cell-free reactions were used for pull-down experiments without further purification.

Linear templates encoding SH2 proteins selected for MST measurements were cloned into vector pIX3.0 via XhoI and BamHI. The generated plasmids were expressed using the RTS500 PM HY kit (5 PRIME GmbH, Hamburg, Germany) overnight at 32°C, 900 rpm. The *amber* stop codon was suppressed with 200 nM tRNASerCUA [51], a tRNA aminoacylated by endogenous seryl-tRNA-synthetase. Synthesized proteins were partly purified by buffer exchange to PBS (Carl Roth, Germany) via a NAP-10 column (GE Healthcare) and ultracentrifugation at 264,000× g, 90 min at 4°C (Beckman Coulter). Supernatants containing fully soluble SH2 proteins were used for MST measurements without further treatment.

### Synthesis of phosphorylated ADAP and YEEI fusion proteins

For the synthesis of SNAP-tagged ADAP, the sequence coding the section 570-690 was cloned into the SNAP-tag expression vector pSET7-26b (NEB) via BamHI, XhoI. The sequence of the middle T-antigen peptide SNAP-YEEI was custom synthesized by GeneArt (Regensburg, Germany) and cloned into pSET7-26b via BglII and EcoRI sites. Both proteins were synthesized using the EasyXpress Protein Synthesis Kit Mini (Qiagen, Germany) with the supplementation of 15 µM SNAP-specific BG-647 dye (NEB) for 60 min at 37°C and 500 rpm.

For *in vitro* phosphorylation, the proteins were re-buffered into phosphorylation buffer (50 mM Tris/HCl pH 7.5, 150 mM NaCl, 9.5 mM MgCl_2_, 0.5 mM MnCl_2_, 2 mM EGTA, 0.4 mM Na_3_VO_4_, 2 mM DTT and 4 mM ATP). Recombinant Fyn-kinase [24] was added at a molar ratio of 1:20. The phosphorylation reaction was performed over night at RT and subsequently stopped by purification on Ni-NTA magnetic agarose beads (Qiagen, Germany). The eluted proteins were buffer exchanged into 1× HBS (50 mM HEPES, 150 mM NaCl, pH 7.6) and were stable for at least 6 months at 4°C. For non-phosphorylated controls, the synthesized proteins were directly purified and re-buffered in HBS.

### Procedure of pull-down experiments

25 µL reaction containing freshly synthesized, site-specifically biotinylated SH2 protein were diluted with 275 µL of 1× PBS (Carl Roth) and incubated with 60 µL streptavidin-coated Dynabeads (Invitrogen, USA) at RT under gentle agitation for 2 h on a stirring wheel. Crude mixtures containing the beads with bound SH2 were split to 2× 150 µL fractions into new vessels and subjected with diluted ADAP in 1× PBS buffer at a molar ratio of approx. 1:3 (~ 4-8 pmol ADAP) to a final volume of 250 µL. The complex was allowed to form for 90 min at RT under gentle agitation. After removal of supernatant and two washes with 100 µL PBS, the beads were boiled in 30 µL SDS-PAGE loading buffer (1× RotiLoad, Carl Roth, Germany) at 90°C for 5 min in order to liberate all immobilized proteins (stripping). The supernatant and wash fractions were subjected to acetone precipitation and redissolved in 30 µL SDS-PAGE loading buffer. The protein fractions were then separated by SDS-PAGE (15% acrylamide). Prior to Coomassie staining, the gel was scanned on a Typhoon imager (Amersham, Fluorescence Mode, excitation 633 nm, 300 V) to detect SNAP-mediated fluorescence signals. Signal intensities were quantified using ImageQuant software. The amount of ADAP bound to the individual SH2 protein was normalized to the total signal intensities of all collected fractions.

### Procedure of supernatant depletion assays

For the performance of pull-down titrations, the SH2 domains were site-specifically biotinylated and charged on streptavidin-coated DynaBeads, as for the pull-down experiments, however with increased volumes (60 µL reaction on 180 µL bead suspension). The proteins were radiolabeled for exact determination of concentration by adding ^14^C-labelled leucine to the synthesis reactions as a tracer. After immobilization for 90 min, the coated beads were washed once with 100 µL 1× PBS and resuspended in 40 µL 1× PBS. Thereof, 20 µL bead suspension was serially diluted into 20 µL 1× PBS. Then, 10 µL of ADAP (diluted in PBS with 0.1% (v/v) *E. coli* extract of the EasyXpress Mini Kit, Qiagen, Germany) was added to a final concentration of 8 nM. A reference without beads was included. The complex was allowed to form for 10 min at RT. Beads were collected and supernatants containing unbound ADAP were acetone precipitated and separated by SDS-PAGE. ADAP was detected and analyzed by fluorescence readout applying the same settings as for the pull-downs. The obtained signal intensities were subtracted from the reference (set as 100% free ADAP) to obtain the degree of bound ADAP for each sample and plotted against the respective concentrations of the SH2 domain. Binding curves were generated in a Microsoft Excel sheet; the K_d_ values were read out directly from the plot.

### Procedure of MST measurements

MST measurements were carried out as described before [52]. The SH2 proteins were serially diluted over close to five orders of magnitude in 1× PBS supplemented with 1.75% (v/v) *E. coli* extract of the EasyXpress Mini Kit as a blocking agent in 200 µL PCR tubes. Afterwards, ADAP was added from a diluted sample in the same buffer to a final concentration of 12 nM, and the mixture was homogenized by 10 times pipetting up and down. The samples were allowed to incubate at RT for 5 min before being loaded into standard-treated Monolith TM capillaries distributed by the manufacturer. After placing into the instrument (Monolith NT.115, NanoTemper, Munich, Germany), the samples were measured by standard protocols. The changes of the fluorescent thermophoresis signals were plotted against the concentration of the serially diluted SH2 proteins. K_d_ values were determined using the NanoTemper analysis software. For exemplary binding curves of each SH2 protein with ADAP-P, please refer to Figure S5. For MST measurements of YEEI, 0.05% Tween-20 (v/v) was added to the interaction buffer, otherwise the samples were processed equally.

## Results and Discussion

### Design of the applied work-flow

**Figure 1:**
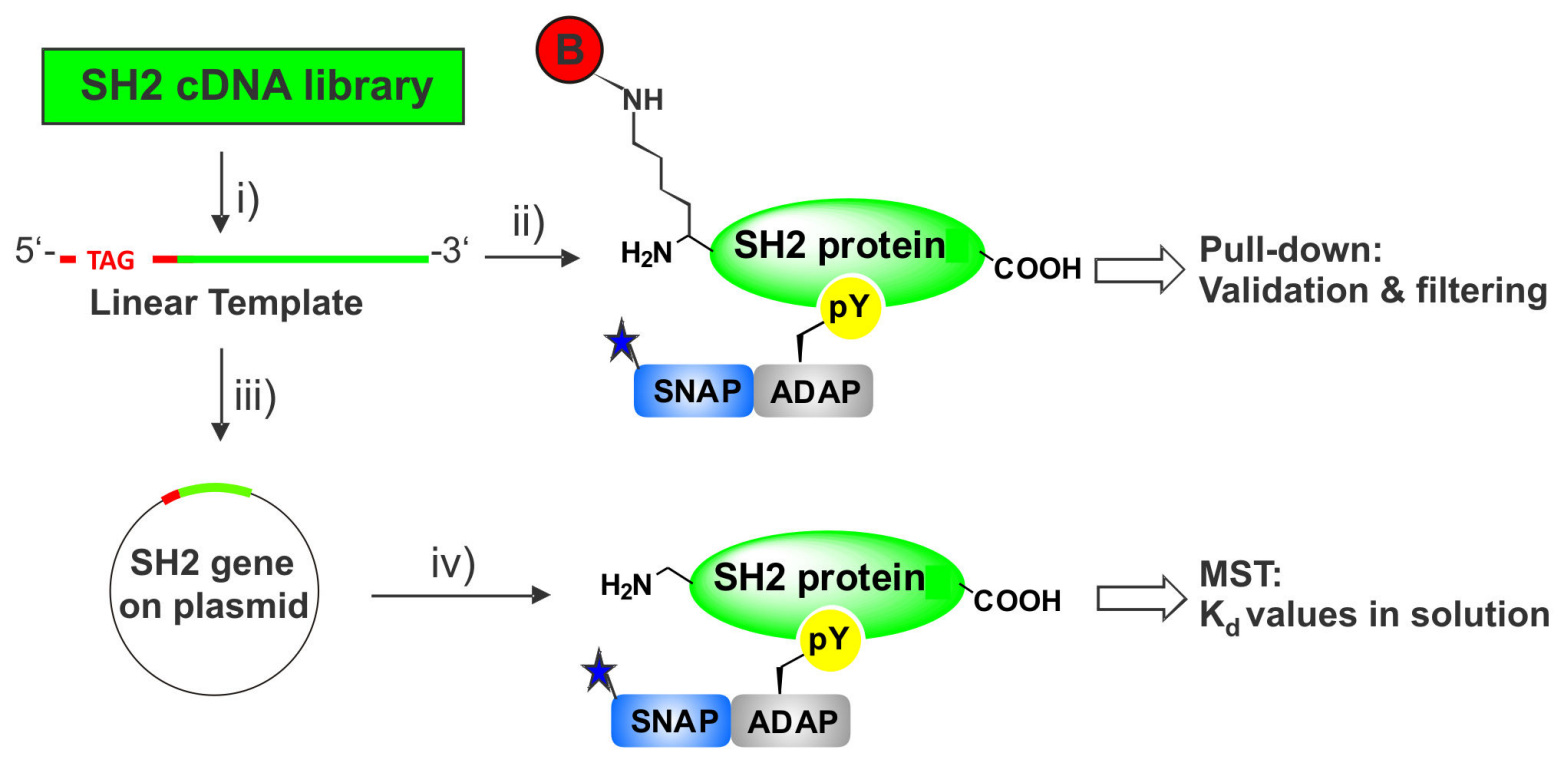
Overview of the applied work-flow for the characterization of SH2-ADAP interactions. i) Amplification of Linear Templates encoding different SH2 proteins by two steps of PCR. ii) Synthesis of site-specifically biotinylated proteins. Subsequent immobilization on streptavidin-coated magnetic beads and pull-down with ADAP for validation and filtering of direct interactions. iii) Cloning of selected Linear Templates into the expression vector pIX3.0. iv) High yield synthesis of SH2 proteins without biotin and subsequent K_d_ value determination using Microscale Thermophoresis.

We intended to characterize the ADAP/SH2 interaction using the following work-flow (Figure 1): First, pull-down experiments with immobilized biotinylated SH2 proteins were performed to validate freshly synthesized interaction partners and to distinguish direct from indirect ADAP interactors. In combination with supernatant depletion, i.e. an expansion of the pull-down method [15,16], pull-downs were also used to confine the number of contemplable interactions and to perform a ranking of interactions based on relative affinities. Then, quantitative interaction data were obtained by applying MST to selected SH2 proteins, receiving K_d_ values in solution.

The phosphoprotein ADAP was synthesized as a fusion protein with the SNAP-tag that was originally developed as a tool for protein labeling in cells [17,18]. We used the SNAP-Tag because it allowed us to perform an efficient and directed co-translational fluorescence labeling far away from the phosphorylation sites. Phosphorylation was performed *in vitro* with recombinant Fyn kinase.

Our time-saving approach comprises the use of small amounts of the fluorescence-labeled interaction partner for all analysis steps, semi-quantitative pull-down, supernatant depletion and proper K_d_ measurement by MST. This allows direct correlation of the obtained results, which is especially desirable because the phosphorylation state of proteins after kinase-mediated *in vitro* phosphorylation is often not fully controllable. In addition, the pull-down can be used as a quality control for the proteins used for quantitative analysis. Both, the fluorescence labeling of ADAP and the uniform biotinylation of cDNA library SH2 proteins significantly increases the detection limit.

A further crucial element of our approach was to synthesize the SH2 proteins for pull-down and supernatant depletion experiments via Expression PCR (E-PCR) [19] from a cDNA generated vector collection [41,42]. E-PCR comprises the use of PCR derived linear templates for cell-free protein synthesis without the need of subcloning. Thus proteins can be delivered within two days. During PCR, a 5’-enchancer sequence was added to the SH2 reading frames containing an *amber* stop codon (TAG) that allows the site-specific incorporation of biotinylated lysine by a chemically charged suppressor tRNA [20]. This eliminated the need for removing free non-incorporated biotin from the reaction mix and permits a directed high affinity immobilization of freshly synthesized proteins on streptavidin-coated magnetic beads without prior purification. Other affinity tags like peptide tags may be used at this step, but we opted for biotin since this provides the highest affinity.

To determine K_d_ values in solution, selected SH2 sequences were subcloned into an expression vector, synthesized in larger scales and subjected to MST measurement. Here, the same fluorescence-labeled ADAP as used previously could be used to generate K_d_ values.

### Synthesis of biotinylated SH2 proteins and *in vitro* phosphorylated ADAP

We decided to validate eight SH2 proteins that were previously identified as potential ADAP interactors by a proteomic approach [30]. Among these were the well characterized interactors Fyn, SLP-76 and NCK2 [23–25], as well as Grap2, PIK3R1, Rasa1, SH2D1A and Lck. Four additional SH2 proteins, not related to the study were also included (Src, Syk, Vav1 and BCAR3), of which Src was expected to interact as it is related to Fyn [43,44], while Vav1 was not because it is known to interact with SLP-76 [45]. The biotinylated SH2 proteins were synthesized by Expression PCR. Here, the biotin moiety was introduced within a constant N-terminal leader sequence in order to enhance protein synthesis from weakly expressed genes and to provide an optimized constant context for biotinylation. The system was designed to produce protein yields in a narrow range which, in the case of biotinylated SH2 proteins, was 20-60 µg per mL reaction corresponding to a molar yield between 0.4 and 2.7 µM depending on the molecular weight (Table 1).

**Table 1:** Yields and sizes of site-specifically biotinylated SH2 proteins.

**Protein**	**Size**	**Yield**	**Yield**
	**[kDa]**	**[µg/ml]**	**[µM]**
Nck2	12.4	33	2.7
Fyn	11.9	24	2.1
Src	13.4	27	2.1
SH2D1A	16.0	31	2.0
Lck	13.2	23	1.8
Grap2	39.8	59	1.5
SLP-76	14.7	23	1.4
Rasa1b	32.9	37	1.2
Syk	32.0	32	1.0
Vav1	18.1	15	0.9
PIK3R1	49.9	35	0,7
BCAR3	93.5	43	0.5
Rasa1	48.2	19	0.4

**Figure 2:**
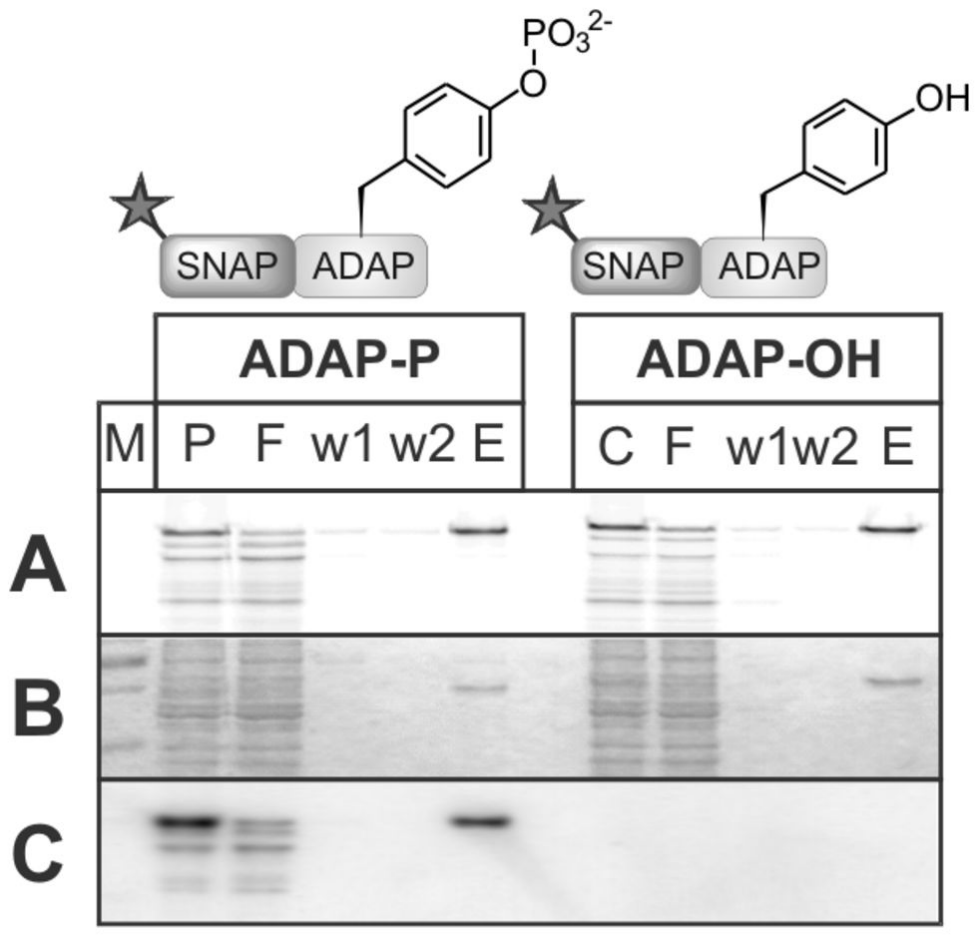
His-Tag purification of phosphorylated ADAP (ADAP-P) and non-phosphorylated ADAP (ADAP-OH). (A) Detection of fluorescent ADAP in SDS gel (excitation 633 nm), (B) Coomassie stain, (C) Western Blot with anti phosphotyrosine antibody. P: cell-free synthesis of ADAP after *in* *vitro* phosphorylation, C: non-phosphorylated ADAP synthesis, F: flow-through, w1 and w2: wash fractions, E: purified ADAP.

After having obtained soluble biotin-tagged SH2 proteins (Figure S1), we generated the tyrosine phosphorylated interaction partner ADAP. As mentioned before, ADAP is multiply phosphorylated *in vivo* by Fyn-kinase in its C-terminal region and reported to promote binding to various SH2 domains [24]. The relevant section of ADAP (amino acids 570-690) was expressed as a fusion protein with the SNAP-tag. Following synthesis, the fluorescently labeled fusion protein SNAP-ADAP (onwards referred to only as ADAP) was phosphorylated *in vitro* by recombinant Fyn kinase and subsequently purified via its His-tag. Phosphorylation was confirmed by anti-phosphotyrosine western blotting and resulted in a lower apparent molecular weight in SDS-PAGE compared to the non-phosphorylated species. This analysis indicates an almost complete phosphorylation (Figure 2). Additional MS analysis confirmed that approx. 90% of each phosphorylation site was modified (Table S1).

### Validation of direct ADAP interactions with the SH2 library

**Figure 3:**
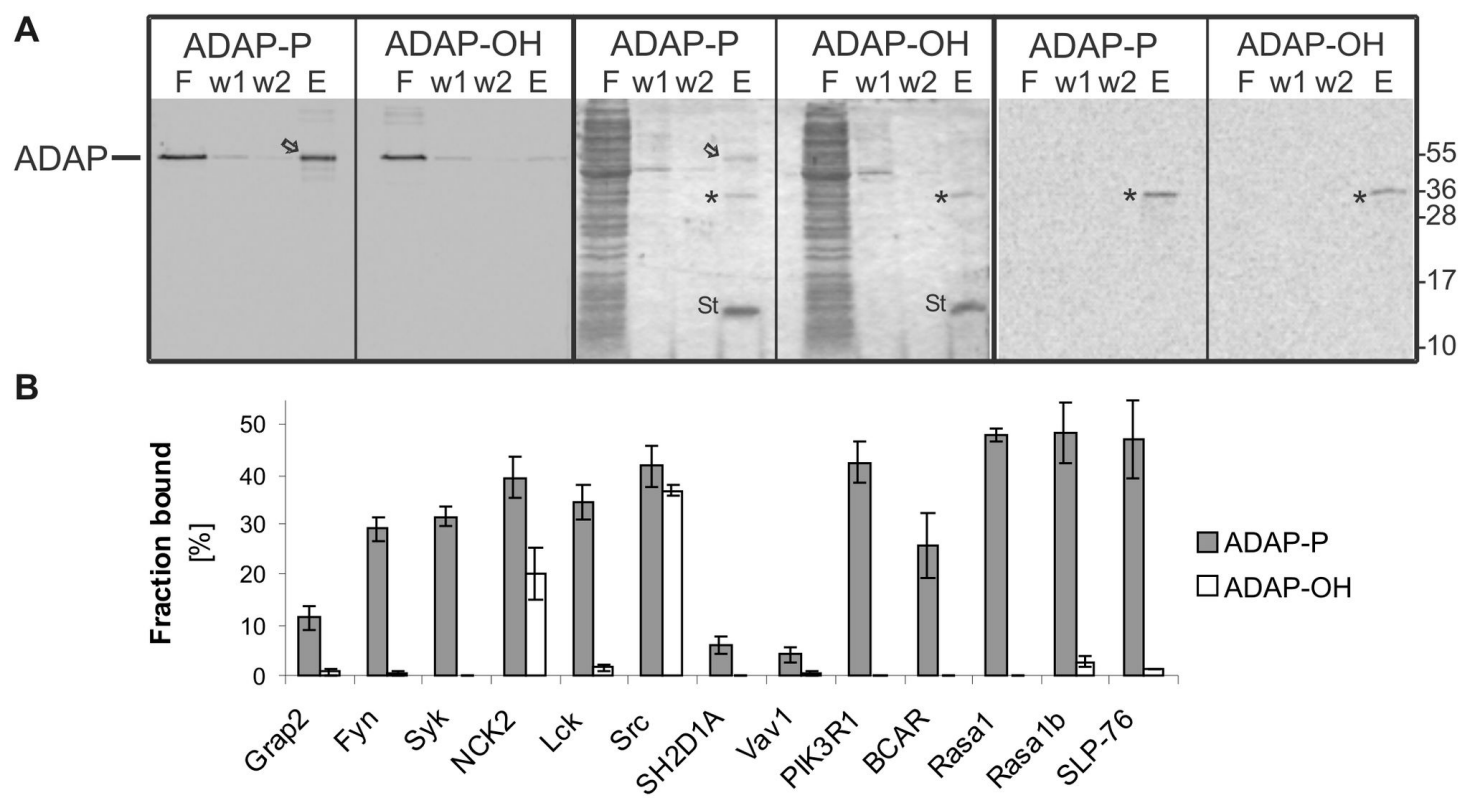
Detection of ADAP-SH2 interactions by pull-down. (A) Pull-down experiment showing the interaction of ADAP and Rasa1b as an example. In this experiment, the SH2 protein Rasa1b was radioactively labeled by adding ^14^C-Leu to the synthesis reaction to allow visualization. Left panels: Detection of fluorescent ADAP in the SDS gel (excitation 633 nm) after electrophoresis, middle panels: Coomassie stain of SDS gel, right panels: Autoradiography of SDS gel with detection of radioactively labeled SH2 protein. ADAP-P: phosphorylated ADAP, ADAP-OH: non-phosphorylated ADAP, F: Flow-through on beads containing unbound ADAP and *E. coli* lysate proteins, w1, w2: wash fractions, E: strip fraction containing liberated complex of ADAP, SH2 and streptavidin. Bound and liberated ADAP is marked by an arrow, Rasa1b is marked by an asterisk, St: streptavidin. (B) results of pull-down screening. gray columns: ADAP-P, white columns: ADAP-OH. Error bars represent triplicates.

Biotinylated SH2 proteins were immobilized on streptavidin magnetic beads for pull-down experiments. This step did not require any preceding treatment or purification step, rapidly accelerating the work-flow. Upon immobilization, phosphorylated ADAP (ADAP-P) was added at a roughly constant ratio to the otherwise unaltered affinity matrix. Non phosphorylated ADAP (ADAP-OH) treated in the same manner served as the negative control. The readout of the pull-down experiments was conducted by SDS-PAGE and in-gel monitoring of the fluorescence as shown in Figure 3A for the interaction between ADAP and Rasa1b.

ADAP shows different degrees of binding to the tested proteins (Figure 3B). ADAP-P showed moderate affinity towards Fyn, Syk and Lck. Reduced affinity was detected against Grap2 and BCAR3 whereas almost no binding was observed towards SH2D1A and Vav1. High signal intensities were obtained in case of PIK3R1, NCK2 and Src. The latter two proteins showed notable binding of ADAP-OH as well. Exemplarily, pull-down samples of Syk, Src and Vav1 with ADAP-P and ADAP-OH were extended to Western blotting with a phosphotyrosine antibody (Figure S2) confirming the expected results.

The highest signals of ADAP-P were observed with SLP-76 and two variants of Rasa1, sequences 3-447 and 174-447 (Figure 3B, sequence coverage see Table S2). As no apparent difference in the binding behavior of the two variants was seen, it can be assumed that the hydrophobic N-terminal tail of Rasa1 (1-157) that is reported to exhibit apoptotic properties upon caspase-3 cleavage [46] does not affect the binding properties against ADAP-P. Rasa1 and PIK3R1 contain two SH2 domains. Pull-down analysis of the two isolated domains showed that in both cases the N-terminal SH2 is the main contributor to the observed interactions (Figure S3).

Altogether, our pull-down results reflected the SH2-mediated interaction of ADAP with the literature known binding partners SLP-76, Fyn and NCK2. In addition, the previously identified putative interactions with Rasa1, PIK3R1, Lck, Grap2 and SH2D1A were verified even though Grap2 binds considerably weaker and SH2D1A shows only marginal affinity. The interactions of ADAP with Src, Syk, and BCAR3 that were so far not detected demonstrate that our qualitative interaction screening approach could help to find new possible interactions.

### Quantitative extension of the pull-down by supernatant depletion assay

To this date, pull-down experiments are usually performed to specifically prove the existence of a binding event in a Boolean fashion. As our detection mode is very sensitive with low amounts of SH2 immobilized, we asked whether the differences in pull-down efficiency may reflect different affinities of ADAP to the respective SH2 domain. Therefore, we decided to extend our pull-down method by the so-called supernatant depletion assay, a straight-forward approach that is well suited to gain quantitative information [15,16]. This assay uses a serial dilution of the immobilized binding partner and a fixed concentration of the free interaction partner (ligand) that is determined in the supernatant at equilibrium. By definition, the concentration of immobilized partner where 50% of the totally added ligand is bound reflects the K_d_. Our biotinylated SH2 domains and the fluorescently labeled ADAP ligand proved to meet the assay requirements very well.

**Figure 4:**
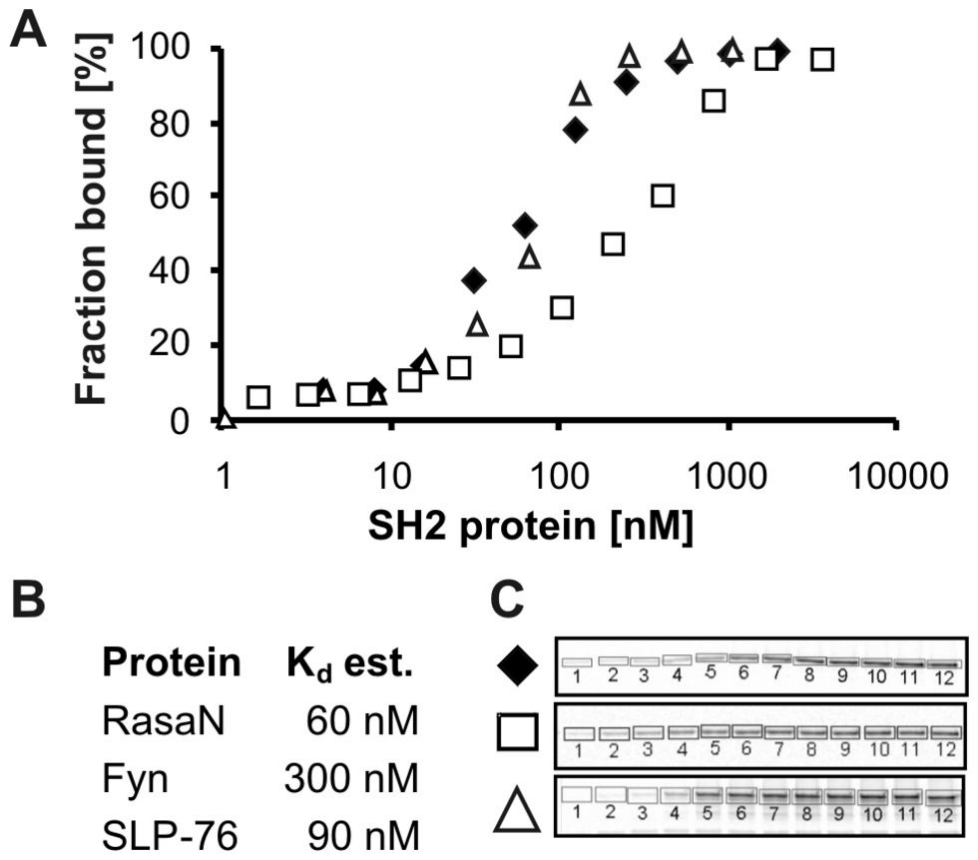
Supernatant depletion assay. (A) Titration of ADAP-P versus selected SH2 domains immobilized on streptavidin magnetic beads. (B) K_d_ values as they were estimated based on the plot shown in A. (C) In-gel fluorescence-detection showing complex depletion, decreasing SH2 concentration from left to right according to increasing numbers. For further explanation please refer to methods. ♦ RasaN, □ Fyn, ∆ SLP-76.

We decided to apply three SH2 proteins to this assay, the strong ADAP binder RasaN and the known binding partners Fyn and SLP-76 [23,25]. As a result we detected interactions of ADAP to both RasaN and SLP-76, with an estimated K_d_ value of 60 to 90 nM (Figure 4). These are very strong interactions within the known range of SH2-mediated interactions of 50 nM to 10 µM [4,37,38]. Fyn showed a K_d_ value of approx. 300 nM, which reflects its lower immobilization degree in the pull-down (Figure 3) but it is still a prominent interacting protein. Control experiments with Fyn and ADAP-OH showed no association, and empty beads did not bind either ADAP-P or ADAP-OH. It should also be noted that the applied concentration of ADAP (approx. 8 nM) is well below the observed K_d_ values, an important requirement for K_d_ determination [15]. For this reason we assume that this type of assay delivers good quality with regard to the establishment of binding hierarchies on solid support.

The set-up of the initial pull-down experiment (Figure 3) comprised SH2 domain concentrations between 70 nM and 140 nM. For this reason, we infer from the supernatant depletion assay that our initial pull-down set-up hit the binding curves at the early slope phase and is able to discriminate between strong and weak ADAP binders. An increase of the SH2 domain concentration would have immobilized all ADAP ligand, possibly culminating in the detection of weak or even unspecific binding events.

### K_d_ determination by microscale thermophoresis

**Figure 5:**
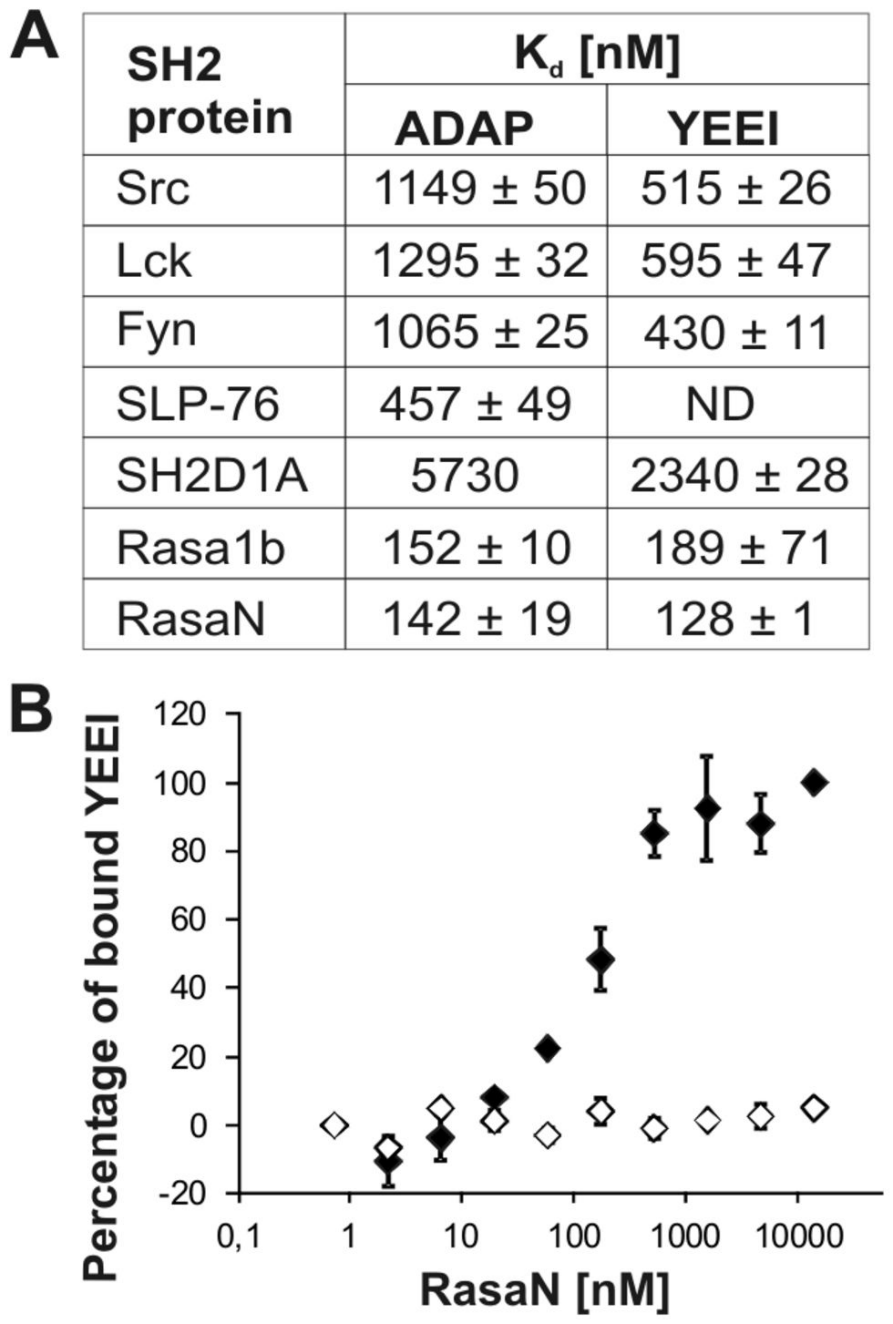
Quantitative interaction analysis. (A) Determined K_d_ values of the interaction of phosphorylated ADAP and YEEI with SH2. Deviations represent duplicates of the measurements. (B) Exemplary MST result for the interaction of phosphorylated YEEI (♦) and non-phosphorylated YEEI (◊) against RasaN. Error bars represent standard deviations of two measurements.

Having obtained semi-quantitative information for various ADAP/SH2 interactions on solid support, we decided to determine K_d_ values in solution for certain SH2 proteins that were selected on the basis of our pull-down results. We included the known ADAP interactors SLP-76 and Fyn, the strongest interactors Rasa1b and its isolated N-terminal SH2 domain (RasaN). Since Fyn is a Src-type kinase, we also included the related SH2 domains of Src and Lck. SH2D1A was included as the weakest binder. In order to obtain the amounts and concentrations of SH2 proteins necessary for MST measurements, the previously used linear templates were cloned and the SH2 proteins synthesized using a dialysis-based preparative cell-free system. The *amber* stop codon in the leader sequence was suppressed with serine instead of biotinylated lysine in order to increase the yield and as MST measurements are conducted using free interaction partners in solution. Yields were up to 100 µM and 2 mg per ml reaction. The SH2 proteins were only partly purified by buffer exchange and ultracentrifugation (Figure S4). We observed different affinities of ADAP to the various SH2 proteins ranging from approx. 100 nM to 5 µM (Figure 5).

The lowest K_d_ values were detected for the interaction with Rasa1. Both, Rasa1b and RasaN bind ADAP with comparable high affinity at around 100 nM, reaching the lower limit for SH2-mediated interactions described in the literature [4,37,38]. The results also indicate that the N-terminal SH2 domain of Rasa1 (RasaN) contributes almost exclusively to the binding. For the known interaction of ADAP to SLP-76, an affinity of around 400 nM was observed. The SH2 domains of the Src-family kinases Src, Lck and Fyn all bind ADAP with similar affinities at around 1 µM. In contrast, SH2D1A showed a 6-fold weaker binding towards ADAP, suggesting the interaction to be less physiologically relevant (Figure S5). All interactions occurred in a phosphorylation-dependent manner and unlike to the pull-down, no interaction was observed for ADAP-OH with the SH2 domain of Src. Altogether the results confirm and extend the qualitative data of our pull-down screening quite well.

In total, the results of the supernatant depletion assay are in accordance with those of the MST measurements. The 2 to 3 times higher affinities of the first method can most likely be attributed to the immobilization of the SH2 domains, creating higher local concentrations on the beads than in solution. Prior to this study no information was available on K_d_ values of ADAP or of phosphoproteins in general. Quantitative data for some of our examined SH2-domains exist only with short solid phase-derived phosphopeptides, most notably with the Y^324^EEI motif of the Middle T Antigen (Hamster Polyomavirus). Its phosphorylation-dependent interaction to Src-SH2 has been widely analyzed including structural [47] and qualitative analysis of the interaction to Fyn, Lck and Src [48]. Moreover, K_d_ values for the pYEEI/Src interaction are reported [49]. In order to further evaluate our system, we decided to expand our study on this well characterized reference sequence.

A small section of the protein (aa 315-331) was fused C-terminally to the SNAP-tag in the same manner as ADAP, applying the same conditions for synthesis, phosphorylation and purification. The new construct combines the specificity of peptides with the properties of proteins. MS analysis of the kinase treated peptide-fusion shows phosphorylation degrees similar to ADAP (Table S1). The results obtained for the phosphorylated YEEI fusion peptide fit accordingly well to the literature. We measured a K_d_ value of approx. 515 nM for the interaction between our YEEI fusion construct and Src (Figure 5), while values between 400 to 700 nM are reported in the literature [49] for the correspondent solid phase peptide, depending on the detection method (SPR or ITC) and tagging of Src-SH2. Additionally, our YEEI construct showed similar affinities towards the Src-SH2 domain and the related domains of Fyn and Lck (as seen in Figure 5), in good agreement with the literature [38].

This supports the viability of the K_d_ values we determined for ADAP. Thus, we were able to establish a binding hierarchy of SH2 proteins to ADAP at a K_d_ level. In accordance to pull-down and supernatant depletion, ADAP shows the highest affinity to Rasa1 (through its N-terminal SH2 domain). This interaction was predicted in SILAC studies [30]. In the context of the observed high affinity this interaction may be of physiological relevance. In contrast, SH2D1A that was predicted as a potential binding partner in the same proteome study was discovered to bind with low affinity.

## Conclusions

We have demonstrated an elegant, effective and fast way to gain qualitative and quantitative information on protein-protein interactions taking full advantage of the potential of cell-free protein synthesis. Independent but complementary methods, all based on the read-out of the same fluorescent binding partner, were used for the analysis: sensitive pull-down, supernatant depletion and MST.

Expression PCR with site-specific biotinylation enables us to get fast access to multiple proteins for pull-down experiments starting from a cDNA vector collection. Subsequently, supernatant depletion was used to rate the pull-down results and to confirm that our pull-down conditions are below the threshold of saturation and can be considered as semi-quantitative. Whereas the first two methods rely on immobilization, MST was used to confirm the pull-down data and to expand them to Kd values in solution. The relative affinities obtained in solution are in accordance with those obtained on solid phase thereby supporting the same binding hierarchy. The overall viability of our MST data is supported as we could reproduce the known binding constant of Src-SH2 to the Middle T Hamster Antigen phosphorylation motif (YEEI) [49].

The merit of our approach is demonstrated by the successful analysis of the phosphotyrosine mediated interaction of the T-cell protein ADAP with SH2 domain containing proteins, the largest ones with sizes between 32,000 and 93,000 Dalton. We were able to establish a binding hierarchy at a K_d_ level with Rasa1 being the strongest binder to ADAP, followed by the known interactors SLP-76 and Fyn. ADAP showed the highest affinity to Rasa1. This interaction was predicted in SILAC studies [30]. In contrast, SH2D1A that was predicted as a potential binding partner in the same proteome study, was discovered to bind with low affinity.

Rasa1 binds ADAP through its N-terminal SH2 with a K_d_ of approx. 100 nM. Thus, this interaction is not only considerably stronger than the interaction of ADAP with its well-known binding partners SLP-76 and Fyn, but also remarkably strong for a pTyr/SH2 interaction in general which are reported to range between 50 nM and 10 µM [50]. The distinct differences of affinities hint at a hierarchy of binding events that in addition will be regulated by local cellular concentrations *in vivo*. The primary function of Rasa1 is to inactivate the Ras protein (being a Ras GTPase activating protein, RasGAP). When considering that Rasa1 is known to interact via its SH2 domains to receptors such as EGFR, adaptors Dok-1 and Dok-2, transcription factor SOCS3 and p190-RhoGAP [32], and that its affinity to several EGFR-derived pTyr motifs ranges between 366 nM and 1473 nM [4], our findings implicate that the interaction of ADAP and Rasa1 might be of physiological importance and are worth of further investigation *in vivo*.

To our knowledge, this work marks the first description of K_d_ values for phosphotyrosine-mediated interactions on the level of larger functional proteins, as opposed to solid-phase generated phosphopeptides used so far. Next to the validation and expansion of existing proteome data, our expression PCR-based generation of site-specifically biotinylated proteins for pull-down is in principle automatable by liquid handling. In regard to the high sensitivity of the combined methods, our approach is particularly suitable for the analysis of hard to obtain proteins. We are hopeful that it is transferable to the analysis of protein-protein interactions in general.

## Supporting Information

Figure S1 **Synthesis of site-specifically biotinylated SH2 proteins Fyn, Lck, Src, NCK2, Vav1, BCAR3, Rasa1, PIK3R1 from linear templates.** Proteins were labelled with ^14^C-Leucine that was added to cell-free reactions as a tracer. (A) Detection of ^14^C-Leu labelled SH2 proteins after exposure to filmless autoradiography. (B) Coomassie staining of the same gels. T: untreated synthesis reaction. S: soluble fraction, supernatant after 10 min at 16,100× g, 4°C.
(TIF)

Figure S2 **Western Blotting on pull-down samples of ADAP against Syk, Vav1 and Src.** An antibody specific for phosphotyrosine (anti-pTyr-IgG-rabbit) was used. The secondary antibody was anti-rabbit HRP-coupled. ADAP-P: phosphorylated ADAP, ADAP-OH: non-phosphorylated ADAP, Lane F: supernatant containing unbound ADAP, w1, w2: wash fractions, E: strip fraction containing liberated complex of ADAP.
(TIF)

Figure S3 **Pull down analysis of the separated SH2 domains of Rasa1 and PIK3R1.** (A) Fluorescent image (excitation 633 nm) from the pull-down of ADAP against RasaN (N-terminal SH2 domain of Rasa1) and RasaC (C-terminal SH2 domain of Rasa1). (B) Fluorescent image (excitation 633 nm) from the pull-down against PIK3R1 (full-length construct) as well as PIK3N and PIK3C. Legend of the lanes shown in A and B, F: supernatant containing unbound ADAP, w: wash fraction, E: strip fraction containing liberated complex of ADAP. (C) Pull-down results of different Rasa1 and PIK3R1 SH2 domains. Values for full length Rasa1 and PIK3R1 were taken from Figure 2A in the main article. (D) Schematic sequence overview of the examined Rasa1 variants.
(TIF)

Figure S4 **Synthesis and purification of SH2 proteins for MST measurements.** Proteins were synthesized using the RTS500 E. coli HY Kit (5PRIME GmbH, Hamburg, Germany). (A) Example SH2D1A. Lanes 1 to 5: synthesis and partial purification by ultracentrifugation. 1: cell-free synthesis reaction at time zero (t = 0), 2: reaction at t = 20 h, 3: supernatant after 10 min at 16,100× g, 4°C, 4: supernatant after buffer exchange to 1× PBS, 5: supernatant after 90 min at 264,360× g and 4°C (removal of ribosomal proteins can be observed). In order to estimate the concentration, a serial dilution of the partly purified protein was compared with a reference standard. (B) Serial dilutions of all proteins used for MST measurements. (C) Concentrations of proteins used for MST measurements. Note: concentrations reflect the state after buffer exchange (implicating 1.4 times dilution) and ultracentrifugation.
(TIF)

Figure S5 **Exemplary binding curves of MST measurements for each examined SH2 protein against phosphorylated ADAP.**
(TIF)

Table S1 **Phosphorylation state of ADAP and YEEI.** The phosphorylation degree of the individual phosphorylation sites was determined by nano LC-MS/MS with LTQ Orbitrap XL mass spectrometer (ThermoFisher), preceeded by in-gel protease digestion. Identified peptides and degrees of phosphorylation are shown.
(TIF)

Table S2 **Sequence coverage of the SH2 proteins examined in this study.**
(TIF)

Table S3 **Gene specific oligonucleotides.** Gene specific oligonucleotides (obtained from IBA, Göttingen, Germany) shown in the table below were used for the 1^st^ step PCR of SH2 proteins, and for the cloning of ADAP. In bold: constant sequences for hybridization with 2^nd^ step oligonucleotides .
(TIF)

## References

[1] Pawson T, Nash P (2000) Protein-protein interactions define specificity in signal transduction. Genes Dev 14: 1027-1047. PubMed: 10809663. 10809663

[2] Seet BT, Dikic I, Zhou MM, Pawson T (2006) Reading protein modifications with interaction domains. Nat Rev Mol Cell Biol 7: 473-483. doi:10.1038/nrm1960. PubMed: 16829979. 16829979

[3] Pawson T, Nash P (2003) Assembly of cell regulatory systems through protein interaction domains. Science 300: 445-452. doi:10.1126/science.1083653. PubMed: 12702867. 12702867

[4] Jones RB, Gordus A, Krall JA, MacBeath G (2006) A quantitative protein Interaction network for the ErbB receptors using protein microarrays. Nature 439: 168-174. doi:10.1038/nature04177. PubMed: 16273093. 16273093

[5] Jadwin JA, Ogiue-Ikeda M, Machida K (2012) The application of modular protein domains in proteomics. FEBS Lett 586: 2586-2596. doi:10.1016/j.febslet.2012.04.019. PubMed: 22710164. 22710164 PMC3413744

[6] Aebersold R, Mann M (2003) Mass spectrometry-based proteomics. Nature 422: 198-207. doi:10.1038/nature01511. PubMed: 12634793. 12634793

[7] Chandramouli K, Qian PY (2009) Proteomics: Challenges, Techniques and Possibilities to Overcome Biological Sample Complexity. Hum Genomics Proteomics 1: 239204. doi:10.4061/2009/239204. PubMed: 20948568. PMC2950283 20948568

[8] Rich RL, Myszka DG (2007) Higher through-put, label-free, real-time molecular interaction analysis. Anal Biochem 361: 1-6. doi:10.1016/j.ab.2006.10.040. PubMed: 17145039. 17145039

[9] Freyer MW, Lewis EA (2008) Isothermal titration calorimetry: Experimental design, data analysis, and probing macromolecule/ligand binding and kinetic interactions. Methods Cell Biol 84: 79–113. PubMed: 17964929. 17964929 10.1016/S0091-679X(07)84004-0

[10] Gift SK, Zentner IJ, Schön A, McFadden K, Umashankara M et al. (2011) Conformational and structural features of HIV-1 gp120 underlying the dual receptor antagonism by cross-reactive neutralizing antibody m18. Biochemistry 50: 2756-2768. doi:10.1021/bi101160r. PubMed: 21351734. 21351734 PMC3088361

[11] Duhr S, Braun D (2006) Why molecules move along a temperature gradient. Proc Natl Acad Sci U S A 103: 19678-19682. doi:10.1073/pnas.0603873103. PubMed: 17164337. 17164337 PMC1750914

[12] Baaske P, Wienken CJ, Reineck P, Duhr S, Braun D (2010) Optical thermophoresis for quantifying the buffer dependence of aptamer binding. Angew Chem Int Ed Engl 49: 2238-2241. doi:10.1002/anie.200903998. PubMed: 20186894. 20186894

[13] Sweeney MC, Wavreille AS, Park J, Butchar JP, Tridandapani S et al. (2005) Decoding protein-protein interactions through combinatorial chemistry: sequence specificity of SHP-1, SHP-2, and SHIP SH2 domains. Biochemistry 44: 14932-14947. doi:10.1021/bi051408h. PubMed: 16274240. 16274240

[14] Yaoi T, Chamnongpol S, Jiang X, Li X (2006) Src homology 2 domain-based high throughput assays for profiling downstream molecules in receptor tyrosine kinase pathways. Mol Cell Proteomics 5: 959-968. doi:10.1074/mcp.T600002-MCP200. PubMed: 16477079. 16477079

[15] Pollard TD (2010) A guide to simple and informative binding assays. Mol Biol Cell 21: 4061-4067. doi:10.1091/mbc.E10-08-0683. PubMed: 21115850. 21115850 PMC2993736

[16] Arasada R, Pollard TD (2011) Distinct roles for F-BAR proteins Cdc15p and Bzz1p in actin polymerization at sites of endocytosis in fission yeast. Curr Biol 21: 1450-1459. doi:10.1016/j.cub.2011.07.046. PubMed: 21885283. 21885283 PMC3350781

[17] Keppler A, Gendreizig S, Gronemeyer T, Pick H, Vogel H et al. (2003) A general method for the covalent labeling of fusion proteins with small molecules in vivo. Nat Biotechnol 21: 86-89. PubMed: 12469133. 12469133 10.1038/nbt765

[18] Keppler A, Pick H, Arrivoli C, Vogel H, Johnsson K (2004) Labeling of fusion proteins with synthetic fluorophores in live cells. Proc Natl Acad Sci U S A 101: 9955-9959. doi:10.1073/pnas.0401923101. PubMed: 15226507. 15226507 PMC454197

[19] Merk H, Meschkat D, Stiege W (2003) Expression-PCR: from Gene Pools to Purified Proteins Within 1 Day. In: Swartz JR Cell-Free Protein Expression. Berlin, Heidelberg, NY: Springer pp. 15-23.

[20] Gerrits M, Strey J, Claußnitzer I, von Groll U, Schäfer F et al. (2007) Cell-free Synthesis of Defined Protein Conjugates by Site-directed Cotranslational Labeling. In: Kudlicki T Katzen F Bennett R Cell-free Expression. Austin: Landes Bioscience pp. 166-180.

[21] Geng L, Raab M, Rudd CE (1999) SLP-76 cooperativity with FYB/FYN-T in the Up-regulation of TCR-driven IL-2 transcription requires SLP-76 binding to FYB at Tyr595 and Tyr651. J Immunol 163: 5753-5757. PubMed: 10570256. 10570256

[22] Raab M, Kang H, da Silva A, Zhu X, Rudd CE (1999) FYN-T-FYB-SLP-76 interactions define a T-cell receptor zeta/CD3-mediated tyrosine phosphorylation pathway that up-regulates interleukin 2 transcription in T-cells. J Biol Chem 274: 21170-21179. doi:10.1074/jbc.274.30.21170. PubMed: 10409671. 10409671

[23] Musci MA, Hendricks-Taylor LR, Motto DG, Paskind M, Kamens J et al. (1997) Molecular cloning of SLAP-130, an SLP-76-associated sub-strate of the T cell antigen receptor-stimulated protein tyrosine kinases. J Biol Chem 272: 11674-11677. doi:10.1074/jbc.272.18.11674. PubMed: 9115214. 9115214

[24] Sylvester M, Kliche S, Lange S, Geithner S, Klemm C et al. (2010) Adhesion and degranulation promoting adapter protein (ADAP) is a central hub for phosphotyrosine-mediated interactions in T cells. PLOS ONE 5: e11708. doi:10.1371/journal.pone.0011708. PubMed: 20661443. 20661443 PMC2908683

[25] da Silva AJ, Janssen O, Rudd CE (1993) T cell receptor zeta/CD3-p59fyn(T)-associated p120/130 binds to the SH2 domain of p59fyn(T). J Exp Med 178: 2107-2113. doi:10.1084/jem.178.6.2107. PubMed: 7504057. 7504057 PMC2191307

[26] da Silva AJ, Li Z, de Vera C, Canto E, Findell P et al. (1997) Cloning of a novel T-cell protein FYB that binds FYN and SH2-domain-containing leukocyte protein 76 and modulates interleukin 2 production. Proc Natl Acad Sci U S A 94: 7493-7498. doi:10.1073/pnas.94.14.7493. PubMed: 9207119. 9207119 PMC23849

[27] Griffiths EK, Krawczyk C, Kong YY, Raab M, Hyduk SJ et al. (2001) Positive regulation of T cell activation and integrin adhesion by the adapter Fyb/Slap. Science 293: 2260-2263. doi:10.1126/science.1063397. PubMed: 11567140. 11567140

[28] Baker RG, Hsu CJ, Lee D, Jordan MS, Maltzman JS et al. (2009) The adapter protein SLP-76 mediates "outside-in" integrin signaling and function in T cells. Mol Cell Biol 29: 5578-5589. doi:10.1128/MCB.00283-09. PubMed: 19667077. 19667077 PMC2756887

[29] Pauker MH, Reicher B, Fried S, Perl O, Barda-Saad M (2011) Functional cooperation between the proteins Nck and ADAP is fundamental for actin reorganization. Mol Cell Biol 31: 2653-2666. doi:10.1128/MCB.01358-10. PubMed: 21536650. 21536650 PMC3133383

[30] Lange S, Sylvester M, Schümann M, Freund C, Krause E (2010) Identification of phosphorylation-dependent interaction partners of the adapter protein ADAP using quantitative mass spectrometry: SILAC vs (18)O-labeling. J Proteome Res 9: 4113-4122 10.1021/pr1003054 20568816

[31] McCubrey JA, Steelman LS, Chappell WH, Abrams SL, Montalto G et al. (2012) Mutations and deregulation of Ras/Raf/MEK/ERK and PI3K/PTEN/Akt/mTOR cascades which alter therapy response. Oncotarget 3: 954-987. PubMed: 23006971. 23006971 10.18632/oncotarget.652 PMC3660063

[32] Pamonsinlapatham P, Hadj-Slimane R, Lepelletier Y, Allain B, Toccafondi M et al. (2009) p120-Ras GTPase activating protein (RasGAP): a multi-interacting protein in downstream signaling. Biochimie 91: 320-328. doi:10.1016/j.biochi.2008.10.010. PubMed: 19022332. 19022332

[33] Eck MJ, Shoelson SE, Harrison SC (1993) Recognition of a high-affinity phosphotyrosyl peptide by the Src homology-2 domain of p56lck. Nature 362: 87-91. doi:10.1038/362087a0. PubMed: 7680435. 7680435

[34] Ettmayer P, France D, Gounarides J, Jarosinski M, Martin MS, et al. (1999) Structural and conformational requirements for high-affinity binding to the SH2 domain of Grb2(1). J Med Chem 42: 971-980 10.1021/jm9811007 10090780

[35] Imhof D, Wavreille AS, May A, Zacharias M, Tridandapani S et al. (2006) Sequence specificity of SHP-1 and SHP-2 Src homology 2 domains. Critical roles of residues beyond the pY+3 position. J Biol Chem 281: 20271-20282. doi:10.1074/jbc.M601047200. PubMed: 16702225. 16702225

[36] Tinti M, Kiemer L, Costa S, Miller ML, Sacco F et al. (2013) The SH2 domain interaction landscape. Cell Rep 3: 1293-1305. doi:10.1016/j.celrep.2013.03.001. PubMed: 23545499. 23545499 PMC4110347

[37] Kaushansky A, Gordus A, Chang B, Rush J, MacBeath G (2008) A quantitative study of the recruitment potential of all intracellular tyrosine residues on EGFR, FGFR1 and IGF1R. Mol Biosyst 4: 643-653. doi:10.1039/b801018h. PubMed: 18493663. 18493663 PMC2811368

[38] Hassan NJ, Simmonds SJ, Clarkson NG, Hanrahan S, Puklavec MJ et al. (2006) CD6 regulates T-cell responses through activation dependent recruitment of the positive regulator SLP-76 CD6. Mol Cell Biol 26: 6727-6738. doi:10.1128/MCB.00688-06. PubMed: 16914752. 16914752 PMC1592849

[39] Tan CS, Bodenmiller B, Pasculescu A, Jovanovic M, Hengartner MO et al. (2009) Comparative analysis reveals conserved protein phosphorylation networks implicated in multiple diseases. Sci Signal 2: ra39 PubMed: 19638616. 19638616 10.1126/scisignal.2000316

[40] Liu BA, Engelmann BW, Nash PD (2012) The language of SH2 domain interactions defines phosphotyrosine-mediated signal transduction. FEBS Lett 586: 2597-2605. doi:10.1016/j.febslet.2012.04.054. PubMed: 22569091. 22569091

[41] Büssow K, Nordhoff E, Lübbert C, Lehrach H, Walter G (2000) A human cDNA library for high-throughput protein expression screening. Genomics 65: 1-8. doi:10.1006/geno.2000.6141. PubMed: 10777659. 10777659

[42] Gerhard DS, Wagner L, Feingold EA, Shenmen CM, Grouse LH et al. (2004) The status, quality, and expansion of the NIH full-length cDNA project: the Mammalian Gene Collection (MGC). Genome Res 14: 2121-2127. doi:10.1101/gr.2596504. PubMed: 15489334. 15489334 PMC528928

[43] Payne G, Stolz LA, Pei D, Band H, Shoelson SE et al. (1994) The phosphopeptide-binding specificity of Src family SH2 domains. Chem Biol 1: 99-105. doi:10.1016/1074-5521(94)90047-7. PubMed: 9383377. 9383377

[44] Resh MD (1998) Fyn, a Src family tyrosine kinase. Int J Biochem Cell Biol 30: 1159-1162. doi:10.1016/S1357-2725(98)00089-2. PubMed: 9839441. 9839441

[45] Ksionda O, Saveliev A, Köchl R, Rapley J, Faroudi M et al. (2012) Mechanism and function of Vav1 localisation in TCR signalling. J Cell Sci 125: 5302-5314. doi:10.1242/jcs.105148. PubMed: 22956543. 22956543 PMC3561853

[46] Widmann C, Gibson S, Johnson GL (1998) Caspase-dependent cleavage of signaling proteins during apoptosis. A turn-off mechanism for anti-apoptotic signals. J Biol Chem 273: 7141-7147. doi:10.1074/jbc.273.12.7141. PubMed: 9507028. 9507028

[47] Waksman G, Shoelson SE, Pant N, Cowburn D, Kuriyan J (1993) Binding of a high affinity phosphotyrosyl peptide to the Src SH2 domain: crystal structures of the complexed and peptide-free forms. Cell 72: 779-790. doi:10.1016/0092-8674(93)90405-F. PubMed: 7680960. 7680960

[48] Dunant NM, Messerschmitt AS, Ballmer-Hofer K (1997) Functional interaction between the SH2 domain of Fyn and tyrosine 324 of hamster polyomavirus middle-T antigen. J Virol 71: 199-206. PubMed: 8985339. 8985339 10.1128/jvi.71.1.199-206.1997 PMC191040

[49] Ladbury JE, Lemmon MA, Zhou M, Green J, Botfield MC et al. (1995) Measurement of the binding of tyrosyl phosphopeptides to SH2 domains: a reappraisal. Proc Natl Acad Sci U S A 92: 3199-3203. doi:10.1073/pnas.92.8.3199. PubMed: 7536927. 7536927 PMC42133

[50] Kaneko T, Joshi R, Feller SM, Li SSC (2012) Phosphotyrosine recognition domains: the typical, the atypical and the versatile. Cell Commun Signal 10: 32-52. doi:10.1186/1478-811X-10-32. PubMed: 23134684. 23134684 PMC3507883

[51] Gerrits M (2001) Funktion und Effizienz von amber-Suppressor-tRNAs in der zellfreien Proteinbiosynthese. PhD thesis, Freie Universität Berlin, Germany.

[52] Jerabek-Willemsen M, Wienken CJ, Braun D, Baaske P, Duhr S (2011) Molecular interaction studies using microscale thermophoresis. Assay Drug Dev Technol 9: 342–353. doi:10.1089/adt.2011.0380. PubMed: 21812660. 21812660 PMC3148787

