# Supplementary figures and images for: Completion of Proteomic Data Sets by Kd Measurement Using Cell-Free Synthesis of Site-Specifically Labeled Proteins
Source: PLoS One. 2013 Dec 10;8(12):e82352. doi: 10.1371/journal.pone.0082352 (PMC3858276; doi:10.1371/journal.pone.0082352)

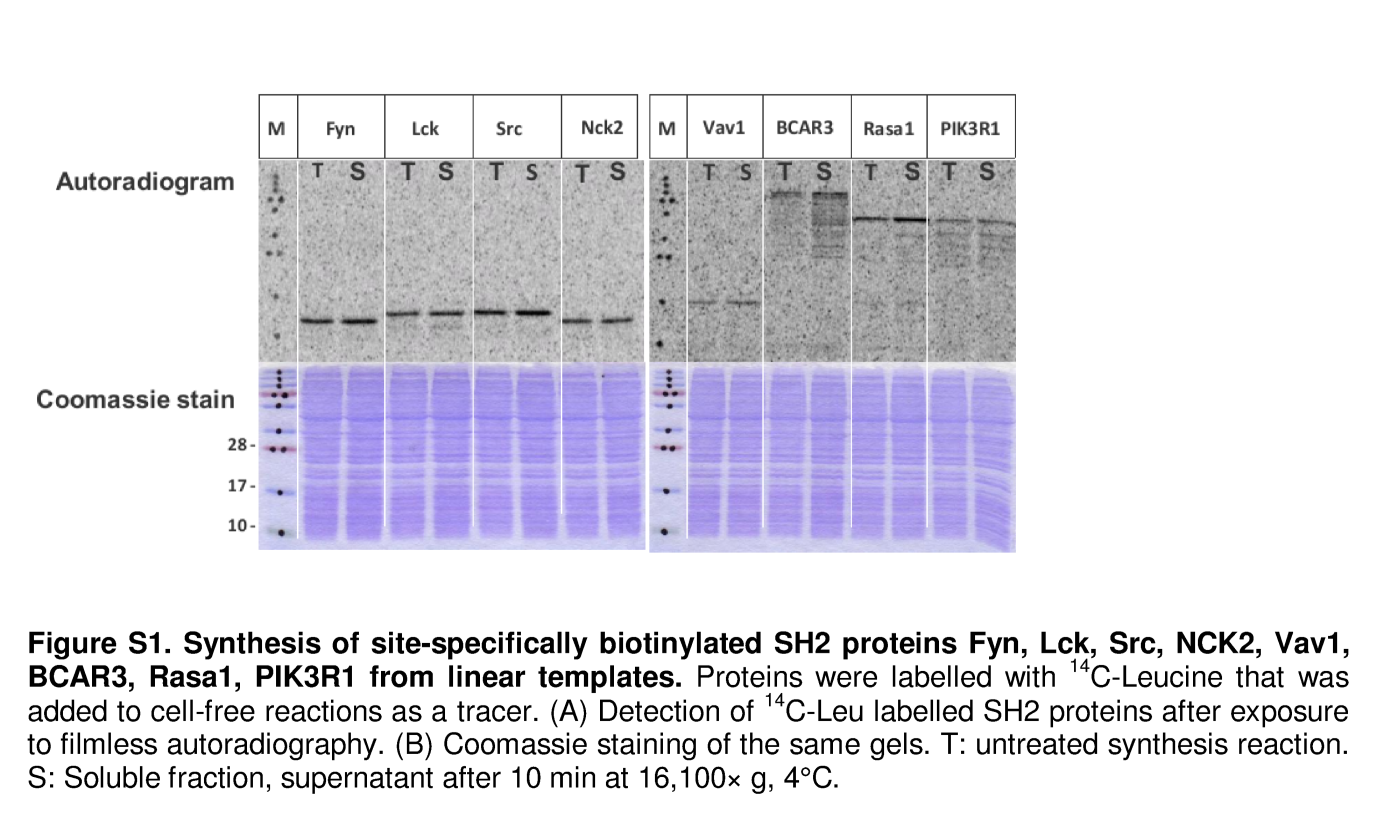

Supplement: Figure S1 — Synthesis of site-specifically biotinylated SH2 proteins Fyn, Lck, Src, NCK2, Vav1, BCAR3, Rasa1, PIK3R1 from linear templates. Proteins were labelled with 14C-Leucine that was added to cell-free reactions as a tracer. (A) Detection of 14C-Leu labelled SH2 proteins after exposure to filmless autoradiography. (B) Coomassie staining of the same gels. T: untreated synthesis reaction. S: soluble fraction, supernatant after 10 min at 16,100× g, 4°C. (TIF) [file pone.0082352.s001.tif]

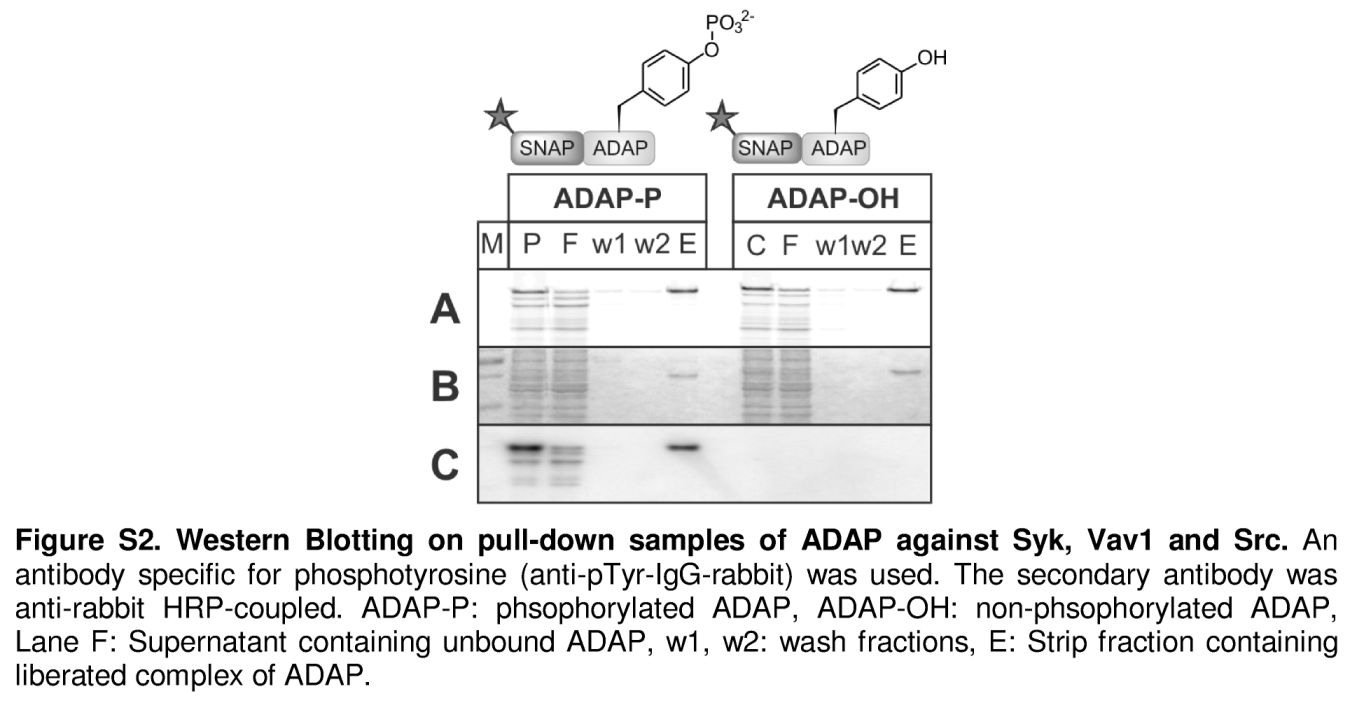

Supplement: Figure S2 — Western Blotting on pull-down samples of ADAP against Syk, Vav1 and Src. An antibody specific for phosphotyrosine (anti-pTyr-IgG-rabbit) was used. The secondary antibody was anti-rabbit HRP-coupled. ADAP-P: phosphorylated ADAP, ADAP-OH: non-phosphorylated ADAP, Lane F: supernatant containing unbound ADAP, w1, w2: wash fractions, E: strip fraction containing liberated complex of ADAP. (TIF) [file pone.0082352.s002.tif]

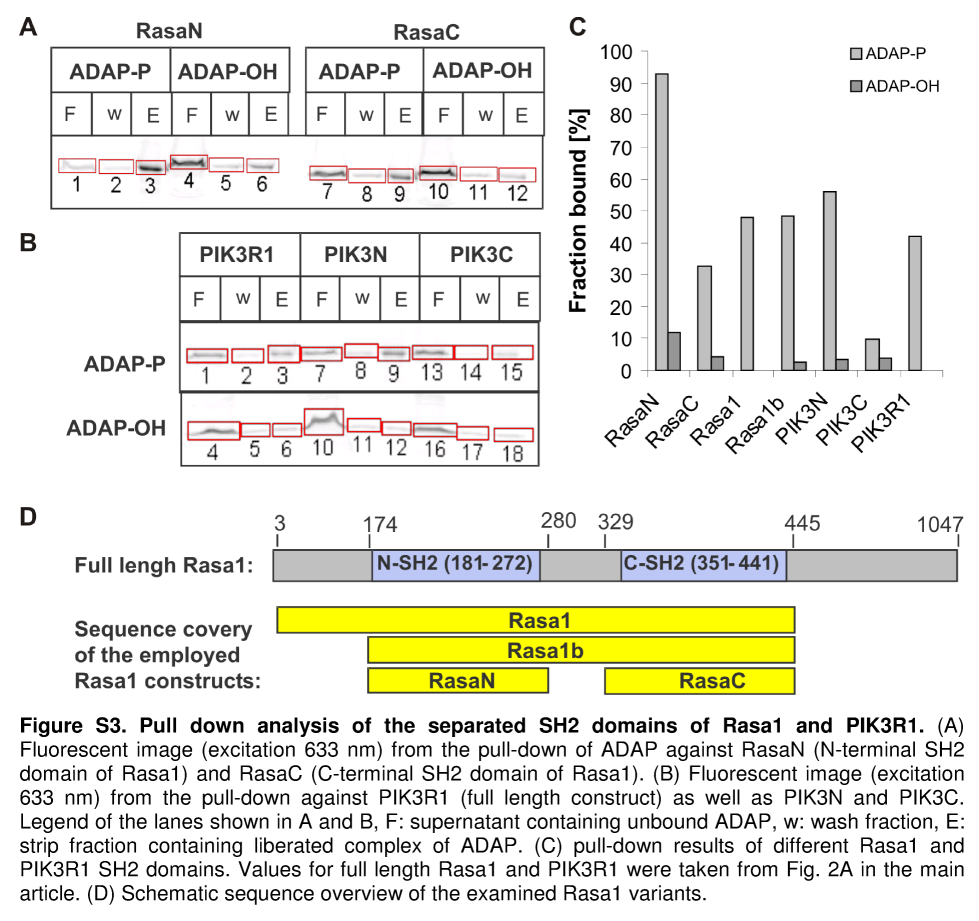

Supplement: Figure S3 — Pull down analysis of the separated SH2 domains of Rasa1 and PIK3R1. (A) Fluorescent image (excitation 633 nm) from the pull-down of ADAP against RasaN (N-terminal SH2 domain of Rasa1) and RasaC (C-terminal SH2 domain of Rasa1). (B) Fluorescent image (excitation 633 nm) from the pull-down against PIK3R1 (full-length construct) as well as PIK3N and PIK3C. Legend of the lanes shown in A and B, F: supernatant containing unbound ADAP, w: wash fraction, E: strip fraction containing liberated complex of ADAP. (C) Pull-down results of different Rasa1 and PIK3R1 SH2 domains. Values for full length Rasa1 and PIK3R1 were taken from Figure 2A in the main article. (D) Schematic sequence overview of the examined Rasa1 variants. (TIF) [file pone.0082352.s003.tif]

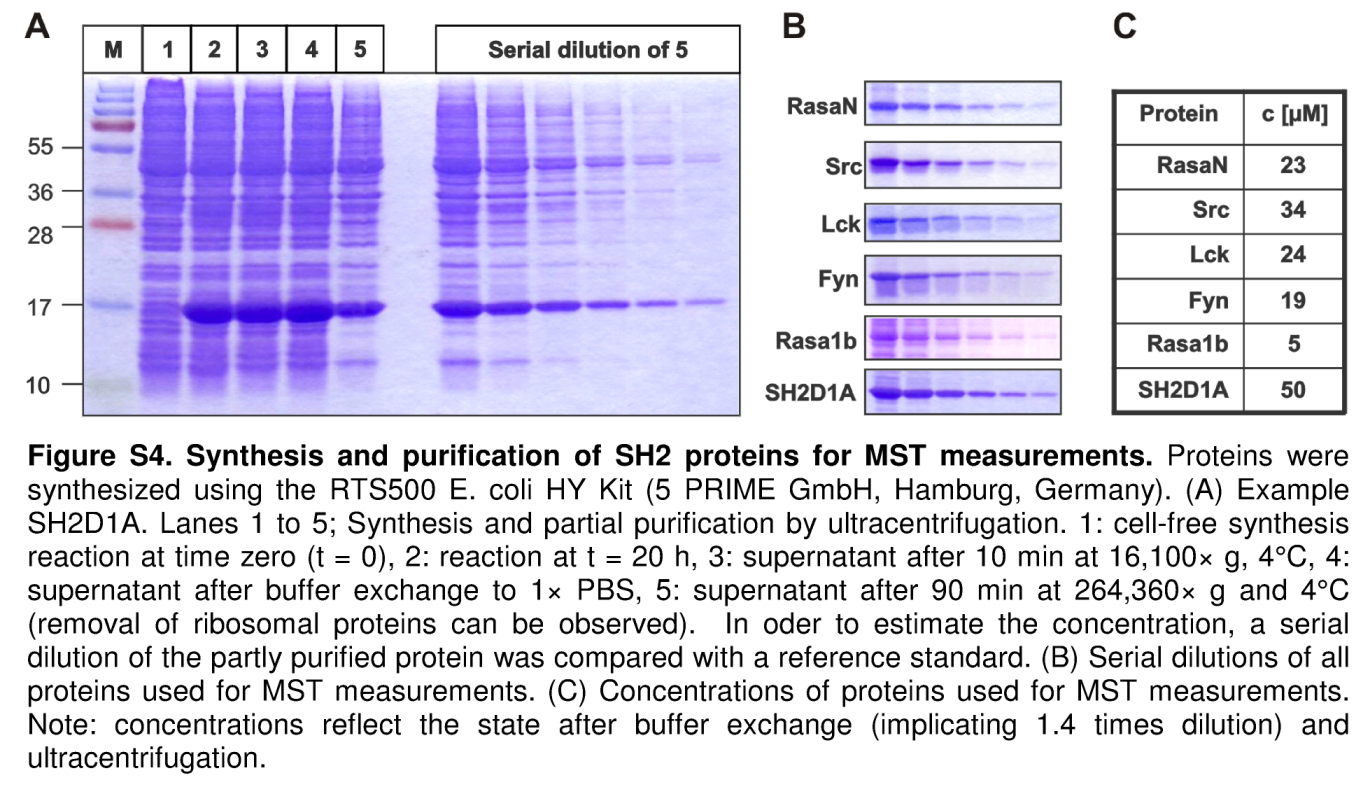

Supplement: Figure S4 — Synthesis and purification of SH2 proteins for MST measurements. Proteins were synthesized using the RTS500 E. coli HY Kit (5PRIME GmbH, Hamburg, Germany). (A) Example SH2D1A. Lanes 1 to 5: synthesis and partial purification by ultracentrifugation. 1: cell-free synthesis reaction at time zero (t = 0), 2: reaction at t = 20 h, 3: supernatant after 10 min at 16,100× g, 4°C, 4: supernatant after buffer exchange to 1× PBS, 5: supernatant after 90 min at 264,360× g and 4°C (removal of ribosomal proteins can be observed). In order to estimate the concentration, a serial dilution of the partly purified protein was compared with a reference standard. (B) Serial dilutions of all proteins used for MST measurements. (C) Concentrations of proteins used for MST measurements. Note: concentrations reflect the state after buffer exchange (implicating 1.4 times dilution) and ultracentrifugation. (TIF) [file pone.0082352.s004.tif]

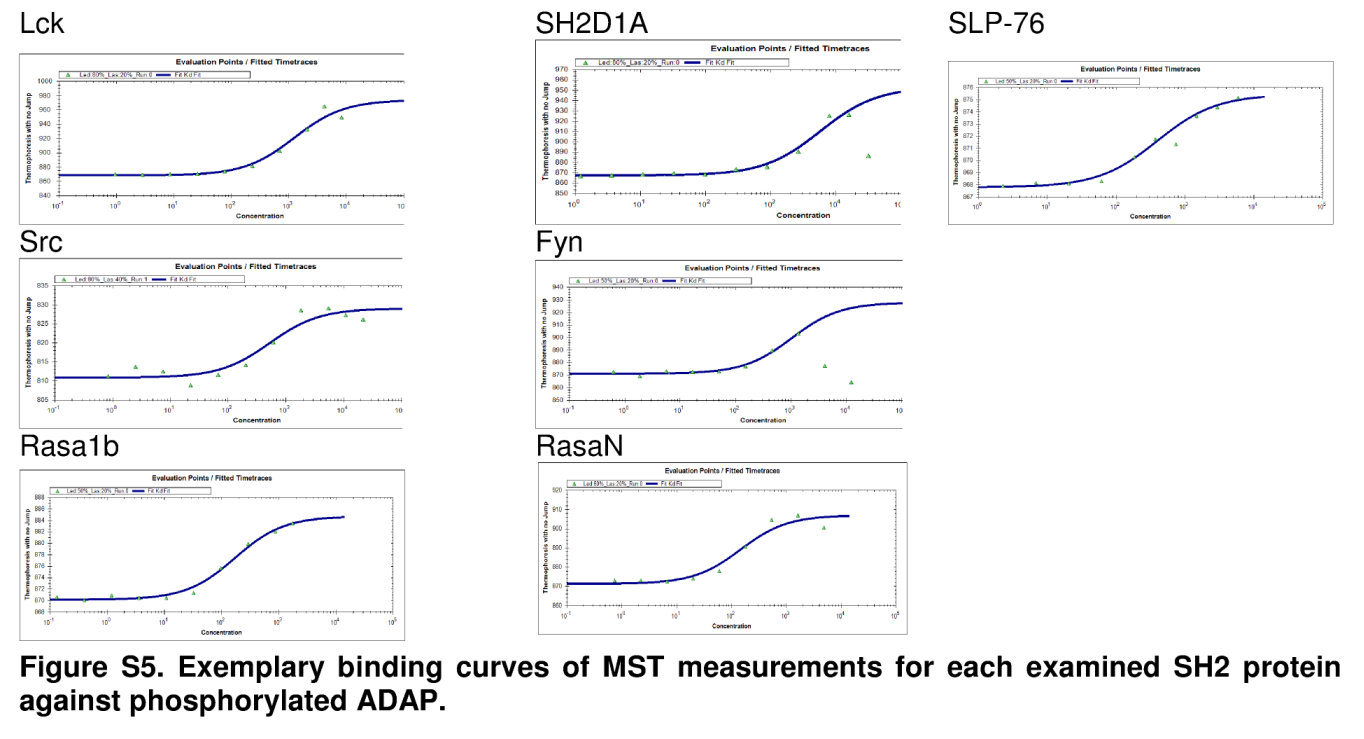

Supplement: Figure S5 — Exemplary binding curves of MST measurements for each examined SH2 protein against phosphorylated ADAP. (TIF) [file pone.0082352.s005.tif]

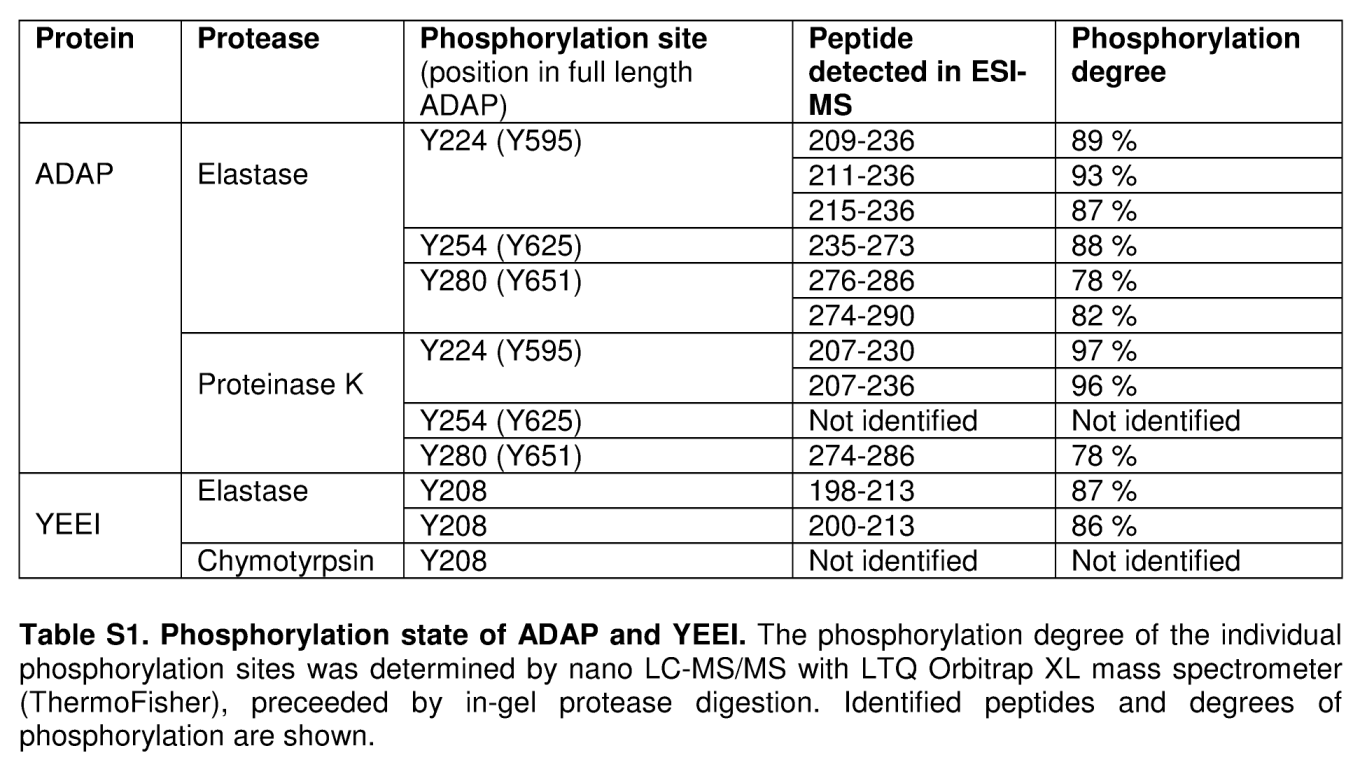

Supplement: Table S1 — Phosphorylation state of ADAP and YEEI. The phosphorylation degree of the individual phosphorylation sites was determined by nano LC-MS/MS with LTQ Orbitrap XL mass spectrometer (ThermoFisher), preceeded by in-gel protease digestion. Identified peptides and degrees of phosphorylation are shown. (TIF) [file pone.0082352.s006.tif]

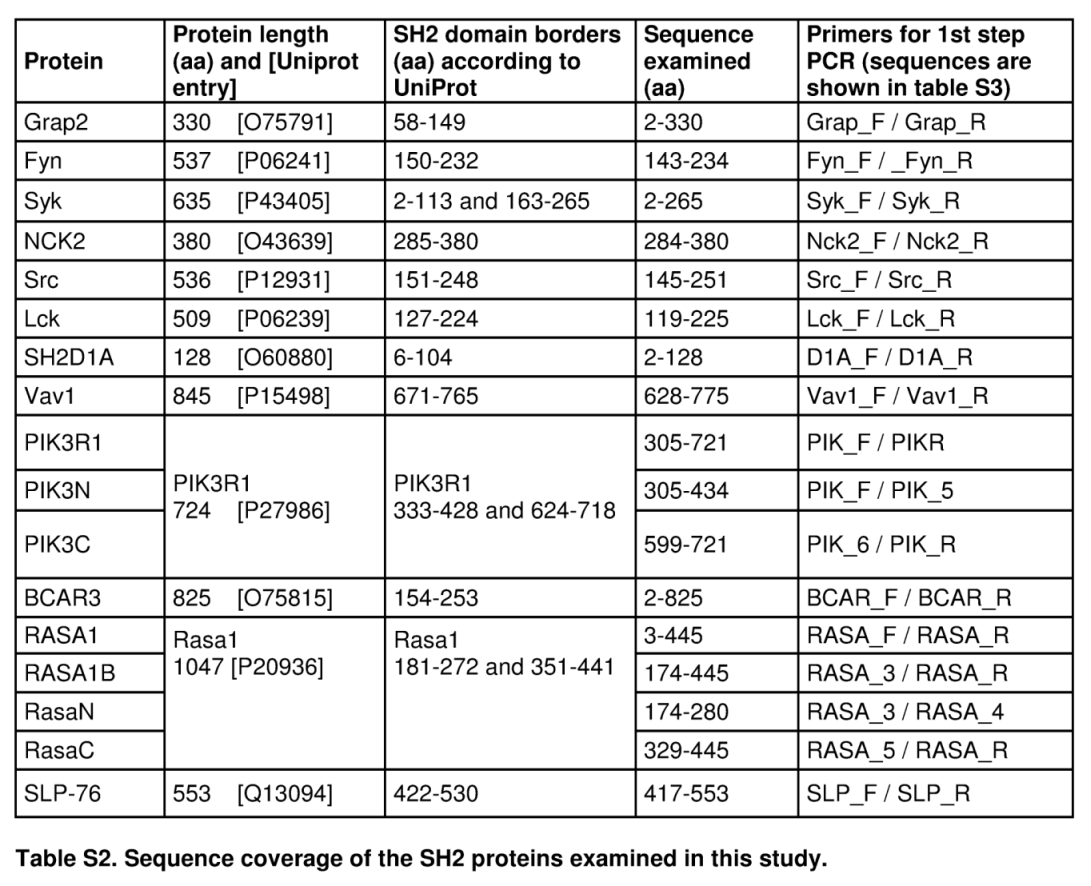

Supplement: Table S2 — Sequence coverage of the SH2 proteins examined in this study. (TIF) [file pone.0082352.s007.tif]

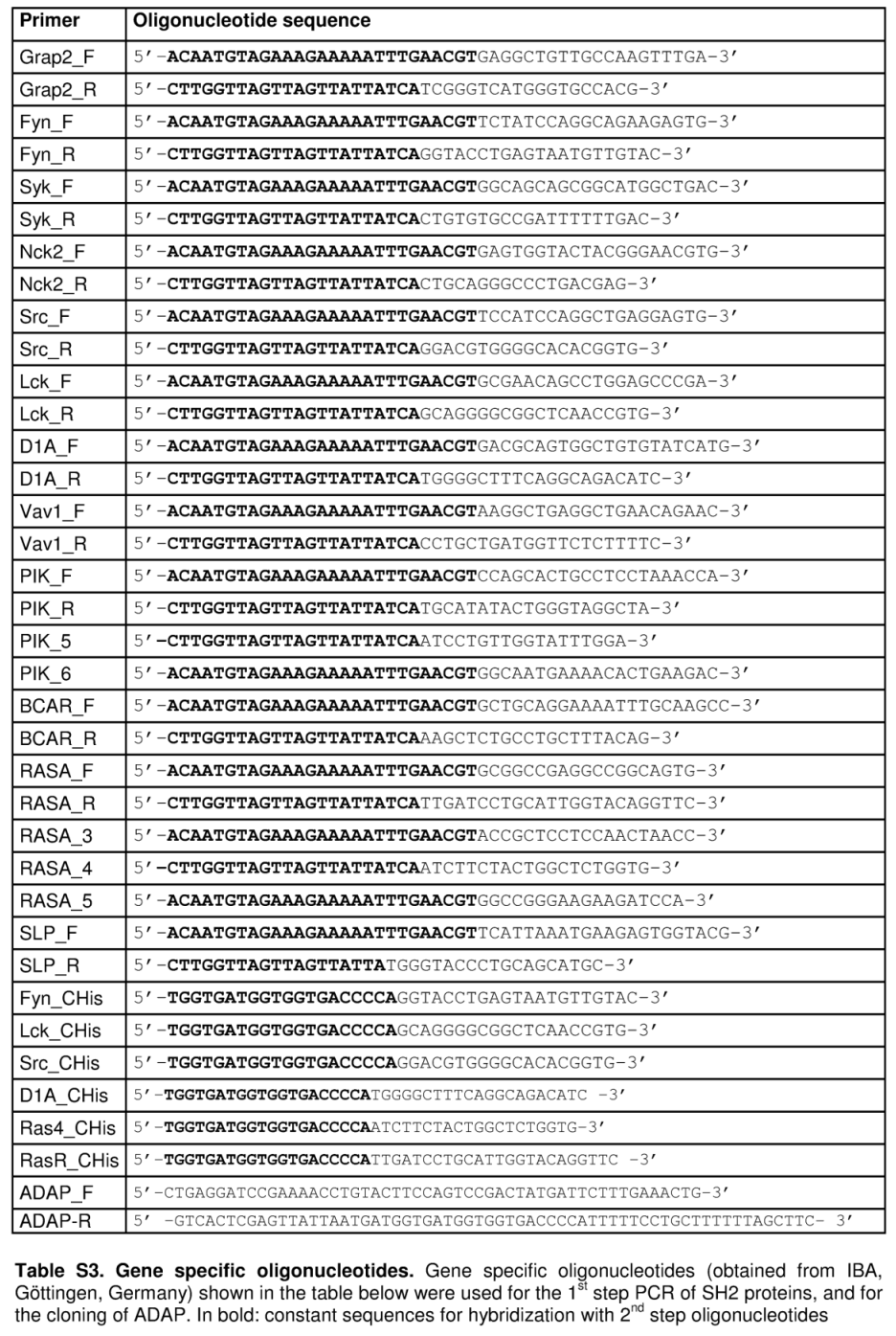

Supplement: Table S3 — Gene specific oligonucleotides. Gene specific oligonucleotides (obtained from IBA, Göttingen, Germany) shown in the table below were used for the 1st step PCR of SH2 proteins, and for the cloning of ADAP. In bold: constant sequences for hybridization with 2nd step oligonucleotides . (TIF) [file pone.0082352.s008.tif]
